# The role of artificial intelligence in the diagnosis and prognosis of traumatic brain injury based on brain CT scans: a systematic review

**DOI:** 10.1007/s10143-026-04476-7

**Published:** 2026-09-12

**Authors:** Eleni Romeo, Petros Baxevanidis, George A. Alexiou, Spyridon Voulgaris

**Affiliations:** 1 Department of Neurosurgery, University of Ioannina, PO BOX 103, Neohoropoulo, Ioannina, 45500 Greece; 2 Medical School, University of Ioannina, Ioannina, Greece

**Keywords:** Traumatic brain injury, Artificial intelligence, Machine learning, Computer tomography, Deep learning, Diagnosis, Prognosis

## Abstract

Traumatic brain injury (TBI) is a leading cause of emergency department visits and a major contributor to injury-related mortality and long-term neurological disability. Non-contrast computed tomography (CT) is the gold-standard imaging modality for the rapid diagnosis of TBI. Clinical outcomes depend strongly on early detection and prompt acute management. Artificial intelligence (AI)–based models may support faster automated identification of traumatic findings and early prediction of patient prognosis. A systematic literature search was conducted in PubMed/MEDLINE, Scopus, IEEE Xplore, ACM Digital Library, and the Cochrane Library in accordance with PRISMA 2020 guidelines to evaluate AI-based models for automated detection of TBI-related findings on CT and for prediction of clinical outcomes. Risk of bias and applicability were assessed using QUADAS-2 for diagnostic accuracy studies and PROBAST + AI for prediction model studies. Twenty-two studies were included. Sixteen studies evaluated diagnostic tasks and 10 evaluated prognostic outcomes, with four studies contributing to both categories. Diagnostic performance was generally high, with many studies reporting AUC values approaching or exceeding 0.90, particularly for larger lesion volumes.Prognostic performance was more variable, with moderate to high discrimination and substantial heterogeneity. Only 9 studies incorporated independent external validation, and performance was frequently lower in external cohorts. All prognostic model studies were judged to be at high overall risk of bias using PROBAST + AI, and most diagnostic accuracy studies also demonstrated high or unclear risk of bias in at least one QUADAS-2 domain, most frequently in patient selection. AI-based models applied to brain CT demonstrate strong technical performance for both diagnostic and prognostic tasks in TBI. However, most studies relied on retrospective designs and lacked independent external validation which limits models generalizability and raises concern for potential overfitting. Prospective, multicenter studies with standardized methodologies and rigorous external validation are required before widespread clinical implementation.

**Supplementary Information:**

The online version contains supplementary material available at https://doi.org/10.1007/s10143-026-04476-7.

## Introduction

Traumatic brain injury (TBI) is a highly heterogeneous neurological condition that represents a leading cause of emergency department visits and remains a major challenge for acute triage and individualized prognosis prediction across the full spectrum of injury severity [1]. According to the World Health Organization (WHO), low- and middle-income countries are responsible for nearly 90% of injury-related deaths worldwide, and an estimated 10 million individuals are affected by TBI each year [2, 3]. In the emergency department setting, non-contrast head Computed Tomography (CT) serves as a fundamental diagnostic and prognostic tool because it is rapid and widely available and can reliably demonstrate time-critical traumatic findings that require urgent management (e.g., intracranial hemorrhage, mass effect, midline shift) [4]. Nevertheless, CT-based assessment is not always straightforward, as subtle lesions, mixed injury patterns, and evolving secondary injury can compromise consistent detection and quantification, while early imaging findings alone are often insufficient to predict the clinical course.

Artificial intelligence (AI), including machine learning and deep learning, has been increasingly explored to support both diagnosis and prognosis in CT-based TBI care. For diagnostic applications, AI models have been developed to identify, classify, segment, and quantify traumatic CT abnormalities [5]. A recent systematic review found multiple clinically relevant TBI-related CT findings that can be automatically identified and quantified with AI, with potential to enhance reproducibility and support clinicians in assessment [6]. For prognostic applications, AI tools aim to predict outcomes such as mortality and functional status (e.g., Glasgow Outcome Scale), and systematic reviews and meta-analyses report generally promising discrimination while highlighting substantial heterogeneity in study populations, endpoints, and validation strategies [7, 8].

In the literature, AI has been evaluated for application to non-contrast head CT in TBI, with previous systematic reviews and meta-analyses reporting generally promising performance for the automated detection and characterization of clinically relevant imaging findings, as well as for selected prognostic tasks [6, 7, 9]. However, reported performance varies with input characteristics, CT acquisition and reconstruction parameters, labeling and reference standards, outcome definitions and time horizons, and validation design in particular [9]. These factors can limit generalizability and complicate comparison between studies, making structured assessment of evidence quality essential. At the same time, across both TBI-focused reviews and broader emergency CT evidence syntheses, several literature gaps are consistently highlighted: (i) heavy reliance on retrospective, single-center datasets and limited external validation, which limits generalizability [6, 7], (ii) substantial heterogeneity in populations, target definitions, labeling standards and endpoints which complicates reliable cross-study comparison and evidence synthesis [6, 7, 9] and (iii) a relative shortage of studies demonstrating real-world clinical impact despite strong stand alone diagnostic and prognostic metrics in some domains [10, 11]. In parallel, prior reviews have noted that reporting in AI medical imaging is often insufficient for clear interpretation and replication, which has led to the introduction of reporting standards oriented to AI and structured checklists to improve transparency and reproducibility.

Accordingly, this systematic review aims to synthesizes studies evaluating AI tools that use brain CT for diagnosis and prognosis of TBI. Specifically, we summarize the evaluated CT findings, prognostic outcomes, model architectures, datasets, and performance metrics, while critically appraising methodological quality, risk of bias, and applicability concerns. By doing so, this review seeks to clarify the current clinical readiness of CT-based AI tools in TBI and to identify evidence gaps and priorities for future validation and model development.

## Materials and methods

This study was designed as a systematic review evaluating the diagnostic and prognostic performance of AI–based tools applied to CT imaging in patients with TBI. The review was conducted in accordance with Preferred Reporting Items for Systematic Reviews and Meta-analyses (PRISMA) 2020 guidelines [12].

### Registration

This systematic review was registered in the International Prospective Register of Systematic Reviews (PROSPERO) under registration number **CRD420251173698**.

### Search strategy

We conducted a systematic search to retrieve relevant studies evaluating the ability of AI tools to assist in the diagnosis and prognosis of TBI based on brain CT imaging. The databases of PubMed/MEDLINE, Scopus, IEEE Xplore, ACM Digital Library and Cochrane Library were searched for retrieve all the available studies. Search strategies were adapted for each database and included combinations of terms related to deep learning, neural networks, traumatic brain injury, CT imaging, diagnosis, classification, and prognosis. Studies published as reviews were excluded using database-specific filters where applicable. The full search strategies for each database are detailed in the supplementary material. The last search in all databases was performed on 15th September 2025.

### Selection process

Two independent investigators (ER and PB) screened independently all retrieved studies by title and abstract and any disagreements were solved by a third reviewer (GAA). Full texts of potentially relevant studies were then assessed against the inclusion and exclusion criteria.

### Eligibility criteria

Eligibility criteria were defined a priori to identify studies evaluating AI-based applications for the diagnosis and prognosis of TBI using computed tomography imaging. The criteria were established to ensure inclusion of relevant human studies assessing AI models applied to CT scans for diagnostic and/or prognostic purposes in traumatic brain injury, while excluding studies outside the defined scope or methodological standards.

#### Inclusion criteria

Studies were eligible for inclusion if they (1) involved human patients diagnosed with traumatic brain injury and evaluated artificial intelligence–based methods applied to computed tomography imaging. (2) Eligible artificial intelligence approaches included machine learning and deep learning techniques, such as convolutional neural networks, recurrent neural networks, transformer-based models, or related architectures. (3) Only studies using computed tomography as the exclusive imaging modality were considered. (4) Studies were required to report diagnostic and/or prognostic performance outcomes of the artificial intelligence models. (5) Original research studies employing retrospective, prospective, cohort, or cross-sectional designs were eligible. (6) Only studies published in English with full-text availability were included.

#### Exclusion criteria

Studies were excluded if they (1) were non-original publications, including reviews, systematic reviews, meta-analyses, editorials, or letters to the editor. (2) Grey literature without peer-reviewed publication, such as conference abstracts, preprints, and theses, was excluded. (3) Case reports or case series involving fewer than 10 patients were not considered. (4) Studies using imaging modalities other than computed tomography, including magnetic resonance imaging, positron emission tomography, or multimodal imaging approaches, were excluded. (5) Additionally, studies that did not involve artificial intelligence–based analysis and relied solely on conventional radiological assessment were not eligible for inclusion.

### Risk of bias and applicability assessment

The risk of bias and applicability concerns of the included studies were assessed independently by two reviewers (ER and PB). For studies evaluating diagnostic accuracy, the Quality Assessment of Diagnostic Accuracy Studies–2 (QUADAS-2) tool was applied [13]. This tool evaluates potential bias across four domains: patient selection, index test, reference standard, and flow and timing. Each domain was judged as having low, high, or unclear risk of bias according to predefined signaling questions.

For studies focusing on prognostic or predictive modeling, the Prediction Model Risk of Bias Assessment Tool (PROBAST + AI) was used [14]. PROBAST + AI assesses risk of bias across four domains: participants, predictors, outcome, and analysis.

Discrepancies between reviewers were resolved through discussion and consensus, with involvement of a third reviewer (GGA) when necessary.

### Data extraction

Data collection was performed using a standardized table that was pre-approved by all authors. The table was independently completed by two authors (ER and PB) to ensure consistency and minimize bias during data extraction. Any discrepancies were resolved by consensus.

The data extraction table included the following variables: study Digital Object Identifier (DOI), first author, year of publication, total number of patients, patient sex distribution, AI model type, model parameters, reference standard (ground truth), dataset partitioning (training, validation, testing, and external validation, when available), diagnostic and prognostic performance metrics (sensitivity, specificity, area under the receiver operating characteristic curve [AUC], and accuracy), clinical task (diagnosis and/or outcome prediction), and additional comments.

### Statistical analysis

Owing to the substantial heterogeneity in both outcome measures and modeling approaches across the included studies, quantitative synthesis was deemed inappropriate and a meta-analysis was therefore not performed. Methodological quality was assessed using the QUADAS-2 tool, implemented in Review Manager (RevMan) version 5.4.1, with results summarized in graphical form.

## Results

### Search findings

After searching the literature, a total of 639 records were retrieved. Specifically, 252, 195, 70, 68, and 54 records were identified through MEDLINE/PubMed, SCOPUS, IEEE Xplore, ACM Digital Library, and the Cochrane databases, respectively. After removal of 157 duplicates, 482 records remained and were screened by title and abstract, resulting in the exclusion of 390 records. The remaining 92 studies were sought for retrieval, of which 86 were successfully retrieved and assessed for eligibility. Twenty-three (23) studies were excluded because they did not include CT scan analysis, four (4) because they did not use AI models, two (2) because they were not related to TBI, four (4) because they were not original research, and thirty-one (31) because they were gray literature studies.

**Fig. 1:**
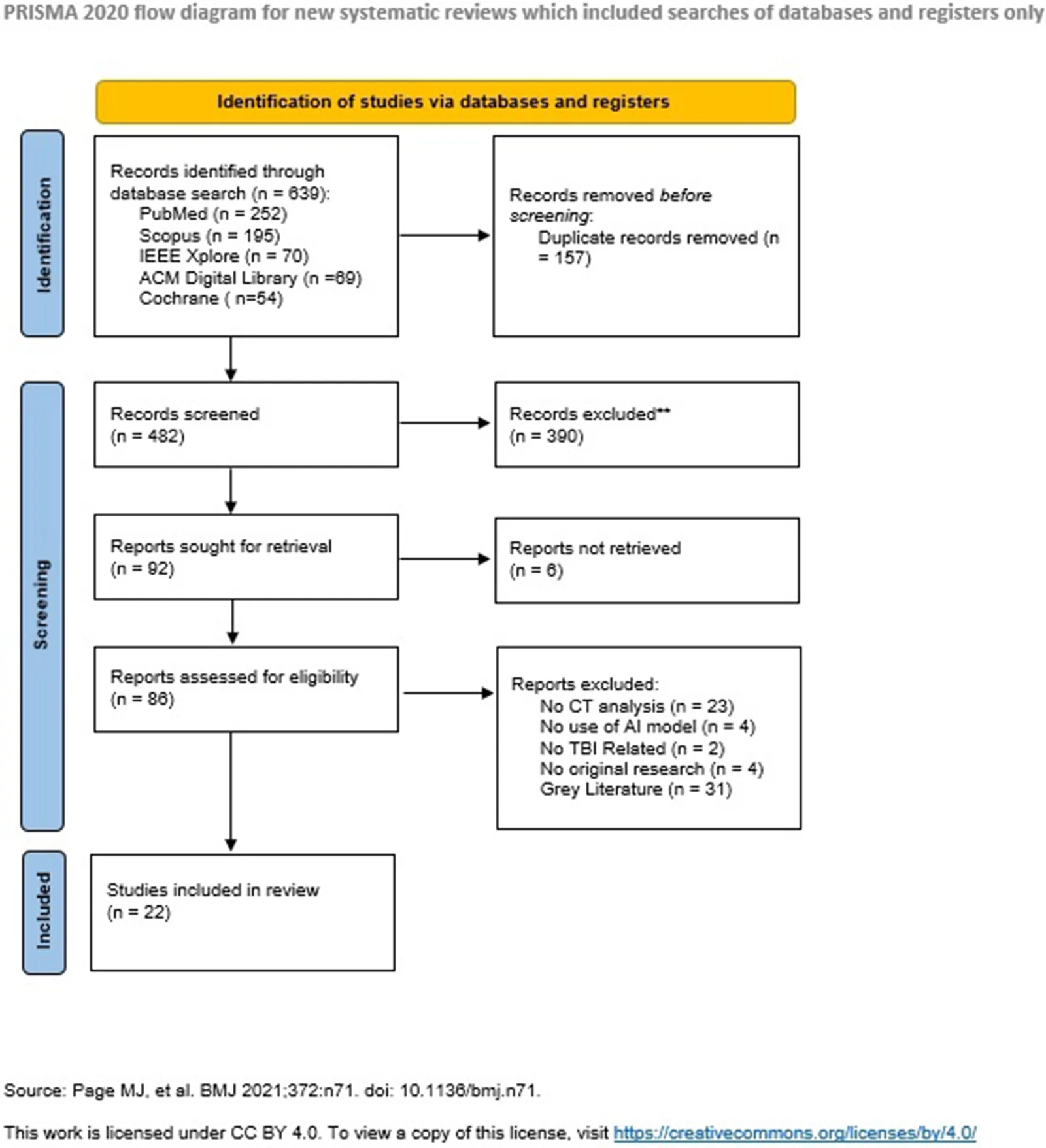
Flow diagram depicting systematic search using PRISMA guidelines

Thus, 22 studies [15–36] were included in our systematic review. The search results and study selection process are presented in a PRISMA 2020 flow diagram shown in Fig. 1.

### Characteristics of the included studies

A total of 22 studies were included in the final qualitative analysis as exhibited in Table 1. Among them, 16 studies evaluated diagnostic tasks and 10 evaluated prognostic outcomes. Four studies contributed to both categories, as they reported both diagnostic and prognostic objectives [17, 24, 25, 28]All included studies investigated patients with ΤΒΙ or AI models intended for TBI-relevant CT findings, and exclusively utilized non-contrast head or brain CT scans as model input. All the studies employed a retrospective observational design, using previously acquired clinical and imaging data. No fully prospective model development studies were identified.

**Table 1:** Characteristic of the included studies

Author	Year	Type of Study	Dataset Size		Model	
			Diagnosis	Prognosis	Diagnosis	Prognosis
Keshavamurthy et al.	2017	Retrospective	409 CT scans	-	SIFT + SVM | CNN + SVM	-
Li et al.	2020	Retrospective	491 TBI patients	-	ResNet50-GRU | ResNet50-LSTM | VGG16-GRU | VGG16-LSTM | VGG19-GRU | VGG19-LSTM | DenseNet121-GRU | DenseNet121-LSTM	-
Yao et al.	2020	Retrospective	120 TBI patients	882 TBI patients	Multi-view CNN + Mixed loss (proposed) | Multi-view CNN + Regular loss | U-Net | ICHNet | 3D U-Net | 3D Active Contour	RF | LR
Monteiro et al.	2020	Retrospective	937 TBI patients	-	DeepMedic (multi-scale 3D CNN)	-
Phaphuangwittayakul et al.	2022	Retrospective	321 CT scans	-	EfficientNet-B2 + DeepMedic	-
Pease et al.	2022	Retrospective	-	537 TBI patients	-	CNN based on AlexNet backbone (imaging) | Linear Discriminant Analysis (LDA) (Clinical Model) | Fusion Model (best performer) | IMPACT-Fusion
Dyer et al.	2022	Retrospective	390 TBI patients	-	EfficientNet-based CNN	-
Angkurawaranon et al.	2023	Retrospective	300 CT scans	-	DeepMedic-based 3D CNN	-
Brossard et al.	2023	Retrospective	-	29 TBI patients	-	CNN1: BLAST-CT (baseline, no retraining) CNN2: 4-class segmentation (fine-tuned BLAST-CT) Manual 4CLASS CNN3: 7-class segmentation (trained from random initialization) CNN4: 7-class segmentation (transfer learning from CNN2) MANUAL 7CLASS
Vidhya et al.	2023	Retrospective	8000 CT slices	-	YOLOv5s-CAM	-
D’Angelo et al.	2024	Retrospective	502 CT scans	-	Dense-UNet	-
Xu et al.	2024	Retrospective	151 TBI patients		DeepLabV3+ | U-Net | ICH-UNet | ResNet-50 | EfficientNet-B6 | ICH-UNet	LR | RF
Puzio et al.	2024	Retrospective	274 TBI patients		3D UNet	
Sato et al.	2024	Retrospective	-	77 TBI patients	-	PY | PO
Hibi et al.	2024	Retrospective	-	1016 TBI patients	-	3D-ViT
Sharma et al.	2024	Retrospective	n/a	-	FedCNN	-
Zhang et al.	2024	Retrospective	-	650 TBI patients	-	MSF
Lin & Yuh	2024	Retrospective	481 Patients	-	PatchFCN (Dilated ResNet-38 backbone) Semi-supervised: Noisy Student	-
Smith et al.	2024	Retrospective	-	2806 patients	-	ViT
Wu et al.	2025	Retrospective	79 CT scans	2228 TBI patients	Keypoint-detection U-Net	MLP
Nagaraju et al.	2025	Retrospective	936 CT slices	-	Combination of: DFA-UNet, GNN, RNN/LSTM, Feature Attention Mechanism, Classification module	-
Emon et al.	2025	Retrospective	82 CT scans	-	UGSS | Sup-ResUNet | Semi-sup CutMix | Semi-sup CutPaste	-

A wide range of AI approaches was utilized across studies. These included traditional machine learning models (e.g. logistic regression, random forests, support vector machines) as well as deep learning architectures, predominantly convolutional neural networks applied to 2D and 3D CT data. Several studies evaluated multiple model architectures or different configurations within the same dataset, which were treated as distinct models when reported separately.

Regarding the clinical objectives, diagnostic studies focused on automated detection, classification, and segmentation of traumatic CT findings, most commonly intracranial hemorrhage and its subtypes, but also including edema, midline shift, mass effect, skull fractures, and related abnormalities. Prognostic studies targeted a broad spectrum of outcomes, including mortality, functional outcome (e.g., GOSE-based endpoints), need for neurosurgical intervention, intensity level therapy, and secondary injury markers (Table 1).

Overall, the included studies demonstrated substantial heterogeneity in study design, dataset characteristics, modeling strategies, and target definitions, which limited direct comparability and supported the decision to perform a narrative rather than quantitative synthesis.

### Risk of bias and applicability assessment

Risk of bias and applicability of the included studies were assessed using the QUADAS-2 tool for diagnostic accuracy studies and the PROBAST + AI tool for prediction model studies. For four (4) studies addressing both diagnostic performance and prediction modelling [17, 24, 25, 28], risk of bias was assessed using both QUADAS-2 and PROBAST + AI, as appropriate.

#### QUADAS-2

A total of 16 studies [15–30] were assessed for the presence of risk of bias in four domains using QUADAS-2. In the patient selection domain, 14 studies were judged to be at high risk of bias [15–22, 24–26, 28–30], while only 2 studies were rated as low risk [23, 27]. For the index test domain, 7 studies were assessed as having low risk of bias [18–21, 23, 27, 30], whereas 9 studies were judged as unclear risk of bias [15–17, 22, 24–26, 28, 29]. Assessment of the reference standard domain indicated low risk of bias in 10 studies [16–21, 23, 26–28] and unclear risk in 6 studies [15, 22, 24, 25, 29, 30]. In the flow and timing domain, 12 studies demonstrated low risk of bias [16–25, 27, 30] while 3 studies were judged to be at high risk of bias [15, 26, 29] and 1 study as unclear risk of bias [28].

Applicability concerns assessed using QUADAS-2 varied across domains. For patient selection, applicability concerns were rated as low in 8 studies [15, 17, 20, 23–25, 27, 30] and high in 8 studies [16, 18, 19, 21, 22, 26, 28, 29]. In the index test domain, 9 studies demonstrated low concern for applicability [16, 18–20, 23–25, 27, 30], while 7 studies were judged as unclear [15, 17, 21, 22, 26, 28, 29]. Applicability concerns related to the reference standard were generally low, with 13 studies rated as low concern [16–23, 26–30] and 3 studies as unclear [15, 24, 25].

**Fig. 2:**
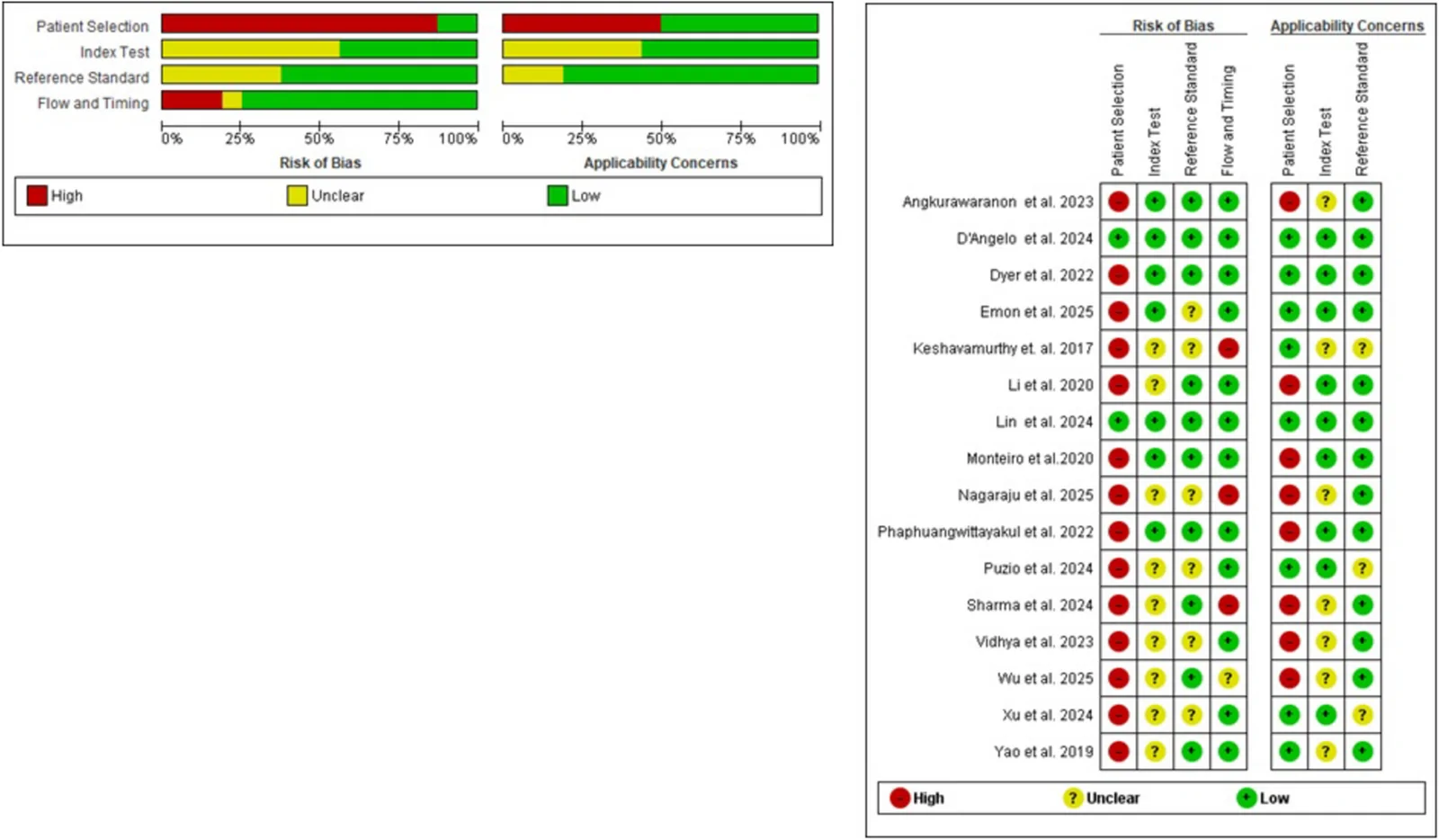
Quadas-2 risk of bias and applicability concerns graph (**A**) and summary (**B**)

The results from the assessment of risk of bias and concerns of applicability are summarized in Fig. 2.

#### PROBAST + AI

Using PROBAST + AI, 10 prediction model studies were assessed [17, 24, 25, 28, 31–36]. Overall, all 10 included prediction model studies were judged to be at high risk of bias. In the participants’ domain, 8 studies were rated as high risk of bias [17, 24, 28, 31–34, 36] and 2 as unclear [25, 35], with no studies assessed as low risk. The predictors domain was predominantly judged as unclear risk of bias 7 [17, 24, 25, 32–34, 36], while 3 studies were rated as high risk [28, 31, 35]. Evaluation of the outcome domain demonstrated mixed risk of bias, with 4 studies rated as low risk [17, 24, 28, 33], 2 studies as high risk [25, 32], and 4 studies as unclear [31, 34–36]. In the analysis domain all 10 studies were judged to be at high risk due to methodological limitations such as inadequate handling of missing data, lack of appropriate validation strategies, potential overfitting, and incomplete reporting of model performance measures.

Applicability assessment using PROBAST + AI showed low concern in 2 studies [31, 34], high concern in 4 studies [17, 28, 33, 35], and unclear concern in 4 studies overall [24, 25, 32, 36]. Applicability concerns were most frequently observed in the participants domain, while predictors and outcomes were generally judged to have low applicability concerns across studies.

**Fig. 3:**
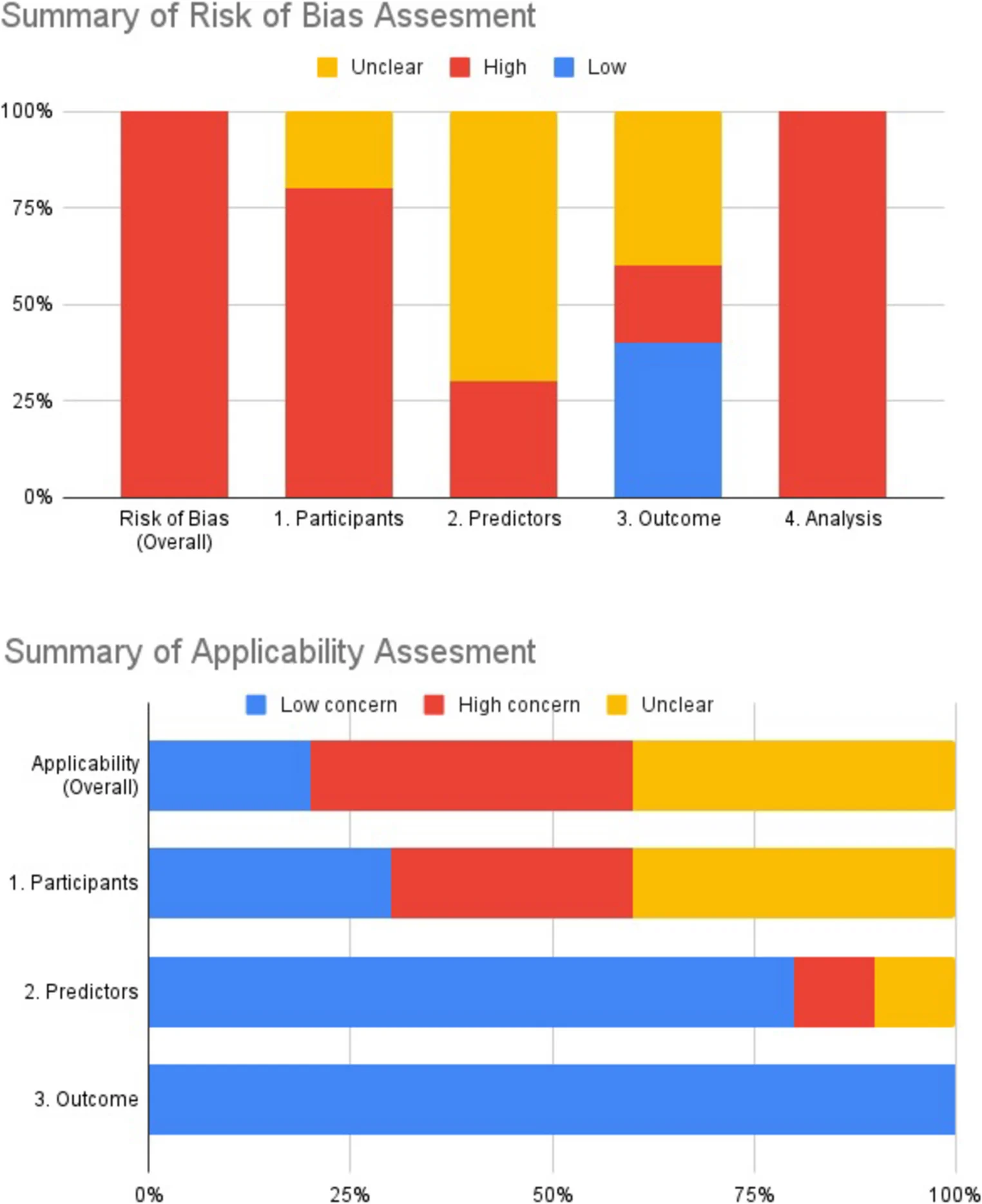
PROBAST + AI probability of bias assessment

The results of PROBAST + AI tool for prediction model studies were summarized in Fig. 3.

### Diagnostic tasks evaluated in CT scan with AI-based models

Among the included studies, 16 evaluated a broad spectrum of diagnostic tasks [15–30] related to traumatic brain injury (TBI) using brain and head CT scans. These tasks encompassed both general abnormality detection and lesion-specific identification, classification, and segmentation.

In three studies, various models were used to assess overall abnormal CT characterization or CT scan usability [16, 18, 26].

The majority of studies [15, 16, 18–23, 26, 27, 29, 30] focused on detection and classification of intracranial hemorrhage and its subtypes. Some studies assessed the presence or absence of intracranial or extra-axial hemorrhage, whereas others developed models to classify hemorrhage subtypes, including epidural hematoma (EDH), subdural hematoma (SDH), subarachnoid hemorrhage (SAH), intraparenchymal hemorrhage (IPH), and intraventricular hemorrhage (IVH). Beyond hemorrhage detection, four (4) studies [16, 18, 20, 23] also evaluated additional traumatic CT findings, including brain edema [18], mass effect [16], midline shift [16, 23], skull fractures [16], and acute infarction [20].

Overall, the included studies demonstrated substantial heterogeneity in diagnostic targets and reported a wide range of performance metrics. The results are summarized in Table 2.

**Table 2:** Diagnostic tasks and performance metrics for models utilized for TBI diagnosis

Diagnostic Tasks	Model	Author	Specific Info	Sensitivity	Specificity	AUC	Accuracy
Abnormal CT characterization	ResNet50-GRU ResNet50-LSTM VGG16-GRU VGG16-LSTM VGG19-GRU VGG19-LSTM DenseNet121-GRU DenseNet121-LSTM	Li et al.		n/a	n/a	0,8934	n/a
	DeepMedic (Multiclass 3D CNN)	Monteiro et al.	ANY LESION - Lesion volume: >0 ml	0,9	0,9	0,89	n/a
			ANY LESION - Lesion volume: >1 mL	0,9	0,9	0,96	n/a
			ANY LESION - Lesion volume: >25 ml	0,9	0,9	0,99	n/a
	FedCNN	Sharma et al.	Overall	0,991	n/a	n/a	0,992
			CQ500	n/a	n/a	n/a	0,987
			RSNA	n/a	n/a	n/a	0,995
			NIH	n/a	n/a	n/a	0,976
ICH Identification-Segmantation-Identification	SIFT + SVM	Keshavamurthy et al.		1	0,79	0,965	0,9
	CNN + SVM	Keshavamurthy et al.		1	0,62	n/a	0,825
	ResNet50-GRU ResNet50-LSTM VGG16-GRU VGG16-LSTM VGG19-GRU VGG19-LSTM DenseNet121-GRU DenseNet121-LSTM	Li et al.		n/a	n/a	0,83	n/a
	Multi-view CNN + Mixed loss	Yao et al.		0,662	0,999	n/a	n/a
	Multi-view CNN + Regular loss	Yao et al.		0,637	0,999	n/a	n/a
	U-Net	Yao et al.		0,619	0,999	n/a	n/a
	ICHNet	Yao et al.		0,72	0,998	n/a	n/a
	3D U-Net	Yao et al.		0,76	0,996	n/a	n/a
	3D Active Contour	Yao et al.		0,489	0,998	n/a	n/a
	Framework with 2 DL branches (EfficientNet-B2 + DeepMedic)	Phaphuangwittayakul et al.		0,958	0,969	n/a	0,962
	Behold.ai (EfficientNet-CNN)	Dyer et al.		0,988	0,925	0,988	n/a
	DeepMedic	Angkurawaranon et al.		0,82	0,9	n/a	0,89
	YOLOv5s-CAM	Vidhya et al.		0,908	n/a	n/a	0,935
	Dense-UNet	D’Angelo et al.		0,9037	0,9485	n/a	0,9124
	ResNet-50	Xu et al.		0,7094	n/a	0,9596	n/a
	EfficientNet-B6	Xu et al.		0,6644	n/a	0,9373	n/a
	ICH-UNet	Xu et al.		0,971	n/a	0,5347	n/a
	PatchFCN (Dilated ResNet-38)	Lin et al.	Baseline	0,92	0,63	0,907	n/a
			Semi-Supervised	n/a	0,804	0,939	n/a
			Picel Oracle	n/a	n/a	0,943	n/a
			QURE.AI	n/a	n/a	0,941	n/a
	Parallel multimodal DL (DFA-UNet + GNN + LSTM)	Nagaraju et al.	DFA-UNet Internal	0,98	0,99	n/a	0,91
			DFA-UNet External	0,994	0,992		0,93
			Proposed multimodal fusion model Batch 16	1	1		1
			Proposed multimodal fusion model Batch 150	0,992	0,99		0,99
	UGSS	Emon et al.		0,751	0,872	0,93	0,8903
	Sup-ResUNet	Emon et al.		0,657	n/a	n/a	n/a
	Semi-sup CutMix	Emon et al.		0,705	n/a	n/a	n/a
	Semi-sup CutPaste	Emon et al.		0,723	n/a	n/a	n/a
EAH (Generally)	DeepMedic	Monteiro et al.	EAH - Lesion volume: >0 ml	0,9	0,9	0,89	n/a
			EAH - Lesion volume: >1 mL	0,9	0,9	0,97	n/a
			EAH - Lesion volume: >25 ml	0,9	0,9	0,99	n/a
	Behold.ai (EfficientNet-CNN)	Dyer et al.		n/a	n/a	0,965	n/a
EDH	Framework with 2 DL branches (EfficientNet-B2 + DeepMedic)	Phaphuangwittayakul et al.		0,955	0,9602	n/a	0,957
	DeepMedic	Angkurawaranon et al.		0,720	0,930	n/a	0,910
	Dense-UNet	D’Angelo et al.		0,9333	0,9979	n/a	n/a
SDH	ResNet50-GRU ResNet50-LSTM VGG16-GRU VGG16-LSTM VGG19-GRU VGG19-LSTM DenseNet121-GRU DenseNet121-LSTM	Li et al.		n/a	n/a	0,92	n/a
	Framework with 2 DL branches (EfficientNet-B2 + DeepMedic)	Phaphuangwittayakul et al.		0,960	0,9755	n/a	0,965
	DeepMedic	Angkurawaranon et al.		0,85	0,82	n/a	0,82
	Dense-UNet	D’Angelo et al.		0,9326	0,9969	n/a	n/a
SAH	ResNet50-GRU ResNet50-LSTM VGG16-GRU VGG16-LSTM VGG19-GRU VGG19-LSTM DenseNet121-GRU DenseNet121-LSTM	Li et al.		n/a	n/a	0,76	n/a
	Behold.ai (EfficientNet-CNN)	Dyer et al.		n/a	n/a	0,973	n/a
	Dense-UNet	D’Angelo et al.		0,9264	1	n/a	n/a
IPH	ResNet50-GRU ResNet50-LSTM VGG16-GRU VGG16-LSTM VGG19-GRU VGG19-LSTM DenseNet121-GRU DenseNet121-LSTM	Li et al.		n/a	n/a	0,69	n/a
	DeepMedic (Multiclass 3D CNN)	Monteiro et al.	IPH - Lesion volume: >0 ml	0,81	0,9	0,87	n/a
			IPH - Lesion volume: >1 mL	0,9	0,9	0,99	n/a
			IPH - Lesion volume: >25 ml	0,95	0,9	0,99	n/a
	Framework with 2 DL branches (EfficientNet-B2 + DeepMedic)	Phaphuangwittayakul et al.		0,958	0,9713	n/a	0,964
	Behold.ai (EfficientNet-CNN)	Dyer et al.		n/a	n/a	0,987	n/a
	DeepMedic	Angkurawaranon et al.		0,83	0,94	n/a	0,93
	Dense-UNet	D’Angelo et al.		0,9186	0,9909	n/a	n/a
IVH	ResNet50-GRU ResNet50-LSTM VGG16-GRU VGG16-LSTM VGG19-GRU VGG19-LSTM DenseNet121-GRU DenseNet121-LSTM	Li et al.		n/a	n/a	0,91	n/a
	DeepMedic (Multiclass 3D CNN)	Monteiro et al.	IVH - Lesion volume: >0 ml	0,9	0,9	0,89	n/a
			IVH - Lesion volume: >1 mL	0.9	0,9	0,99	n/a
			IVH - Lesion volume: >25 ml	-	-	-	n/a
	Behold.ai (EfficientNet-CNN)	Dyer et al.		n/a	n/a	0,976	n/a
	Dense-UNet	D’Angelo et al.		1	1	n/a	n/a
Brain Edema	DeepMedic (Multiclass 3D CNN)	Monteiro et al.	Edema - Lesion volume: >0 ml	0,85	0,9	0,89	n/a
			Edema - Lesion volume: >1 mL	0,9	0,9	0,94	n/a
			Edema - Lesion volume: >25 ml	0,92	0,9	0,98	n/a
Mass Effect	ResNet50-GRU ResNet50-LSTM VGG16-GRU VGG16-LSTM VGG19-GRU VGG19-LSTM DenseNet121-GRU DenseNet121-LSTM	Li et al.		n/a	n/a	0,89	n/a
Midline Shift	ResNet50-GRU ResNet50-LSTM VGG16-GRU VGG16-LSTM VGG19-GRU VGG19-LSTM DenseNet121-GRU DenseNet121-LSTM	Li et al.		n/a	n/a	0,98	n/a
	Dense-UNet	D’Angelo et al.		1	0,855-0,9778	n/a	n/a
Skull Fracture	ResNet50-GRU ResNet50-LSTM VGG16-GRU VGG16-LSTM VGG19-GRU VGG19-LSTM DenseNet121-GRU DenseNet121-LSTM	Li et al.		n/a	n/a	0,86	n/a
Acute Infract	Behold.ai (EfficientNet-CNN)	Dyer et al.		0,833	0,927	0,933	n/a

### Performance metrics of models utilized for TBI diagnosis

Across studies, diagnostic performance was generally high, although substantial heterogeneity was observed on the diagnostic task, lesion subtype, dataset size, and validation strategy.

For overall abnormal CT detection and general lesion identification, reported AUC values were high, with some models approaching or reaching 0.99 [18]. Sensitivity and specificity for general lesion detection and abnormal CT characterization typically ranged from 0.70 to 0.99, with higher performance reported for larger lesion volumes and for models trained on larger datasets [16, 18, 26].

For intracranial hemorrhage detection and subtype classification, performance varied by hemorrhage type. Across EDH, SDH, IPH, and IVH, reported AUC values were high in most studies. Sensitivity and specificity were frequently above 0.90, particularly for deep learning–based detection and segmentation models including Dense-UNet [23], DeepMedic [18], two-branch EfficientNet-B2 + DeepMedic framework [19], and multimodal fusion architecture combining DFA-UNet, GNN, and LSTM [29]. Several studies demonstrated volume-dependent performance, with improved sensitivity, specificity, and AUC for larger lesion volumes (e.g., > 1 mL and > 25 mL) [18].

Reporting diagnostic performance metrics was inconsistent across studies, with some studies [16, 19, 20, 23, 24, 26, 27, 30] not reporting complete sets of sensitivity, specificity, and AUC values for each model, further limiting direct comparison across models and tasks. The results are summarised in Table 2. Moreover, to facilitate clinical interpretation of the diagnostic findings, a summary of the main CT features evaluated across the included studies, together with the best-performing model and best-reported diagnostic performance, is provided in Table 3.

**Table 3:** Summary of clinically relevant CT features and best-reported diagnostic performance

CT feature	Best-performing model	Author	Best-reported performance
Any lesion/abnormal CT finding	FedCNN	Sharma et al.	Sensitivity 99.1%, Accuracy 99.2%
Intracranial hemorrhage (ICH)	Behold.ai (EfficientNet-CNN)	Dyer et al.	Sensitivity 98.8%, Specificity 92.5%, AUC 0.988
Extra-axial hemorrhage (EAH)	DeepMedic	Monteiro et al.	Sensitivity 90.0%, Specificity 90.0%, AUC 0.99 (for lesion volume > 25 ml)
Epidural hematoma (EDH)	EfficientNet-B2 + DeepMedic	Phaphuangwittayakul et al.	Sensitivity 95.5%, Specificity 96.0%, Accuracy 95.7%
Subdural hematoma (SDH)	EfficientNet-B2 + DeepMedic	Phaphuangwittayakul et al.	Sensitivity 96.0%, Specificity 97.6%, Accuracy 96.5%
Subarachnoid hemorrhage (SAH)	Dense-UNet	D’Angelo et al.	Sensitivity 92.6%, Specificity 100%
Intraparenchymal hemorrhage (IPH)	EfficientNet-B2 + DeepMedic	Phaphuangwittayakul et al.	Sensitivity 95.8%, Specificity 97.1%, Accuracy 96.4%
Intraventricular hemorrhage (IVH)	Dense-UNet	D’Angelo et al.	Sensitivity 100%, Specificity 100%
Brain edema	DeepMedic	Monteiro et al.	Sensitivity 92.0%, Specificity 90.0%, AUC 0.98 (Edema lesion volume > 25 mL)
Mass effect	ResNet/VGG/DenseNet + GRU/LSTM	Li et al.	AUC 0.89
Midline shift	ResNet/VGG/DenseNet + GRU/LSTM	Li et al.	AUC 0.98
Skull fracture	ResNet/VGG/DenseNet + GRU/LSTM	Li et al.	AUC 0.86

* The selected model for each CT feature reflects the best overall or most balanced reported performance for that specific diagnostic task, considering sensitivity, specificity, AUC, and accuracy when available

### Models training, validation, and external testing for diagnostic tasks

Training, validation, and testing strategies varied substantially across the included diagnostic studies (Supplementary Table S1). Several studies relied on pretrained models or transfer learning, while others trained models on relatively small datasets.

Most studies relied on internal validation approaches, including random train–validation–test splits and k-fold cross-validation, with limited use of independent external validation cohorts. Several studies employed simple train–test splits, commonly using 80/20 or 60/20/20 proportions for training, validation, and testing [15, 22, 29]. Other studies used k-fold cross-validation (e.g., 5-fold or 10-fold cross-validation) as the primary validation strategy [16, 17, 24]. External validation was reported in a limited number of studies [18–20, 27, 29], which used either separate institutional datasets or publicly available external datasets to evaluate model generalizability.

### Prognostic outcomes tasks evaluated in CT scan with AI-based models

Among the included studies, 10 studies [17, 24, 25, 28, 31–36] evaluated a wide range of prognostic outcomes in patients with TBI using brain/head CT and AI-based models. Prognostic targets encompassed short-term and long-term mortality, functional outcome, neurological deterioration, need for surgical intervention, and markers of disease severity.

Several studies focused on mortality prediction, including 6-month mortality [17, 31], 14-day mortality [24], combined outcomes such as death and coma [24] and overall mortality [28]. These models were frequently based on established clinical prognostic frameworks (such as IMPACT and CRASH models), with AI-based approaches incorporating CT-derived features, volumetric measures, and hemorrhage subtypes to enhance prognostic discrimination.

Functional outcome prediction was also a major focus. Multiple studies evaluated Glasgow Outcome Scale–Extended (GOSE)–based outcomes at different time points, including 3-month [35] and 6–12-month [31, 34] functional status.

Additional prognostic tasks included prediction of need for neurosurgical intervention [25, 36], therapy intensity level [32], and markers of secondary injury [33]. These included prediction of surgery need related to mass effect, high versus low intracranial pressure, and therapy intensity thresholds.

The prognostic outcome task as well as the performance metric for each model are summarized in Table 4.

**Table 4:** Prediction out outcomes task and performance metrics of models that metric the outcome

Prognostics Tasks	Model	Author	Specific Info		Sensitivity	Specificity	AUC	Accuracy
6-month Mortality vs. Survival	LR	Yao et al.	original IMPACT lab model		0,699	0,761	0,798	n/a
			original IMPACT extended model		0,712	0,747	0,795	n/a
			original IMPACT core model		0,704	0,715	0,798	n/a
	RF	Yao et al.	IMPACT lab feature list + Volumes		0,684	0,878	0,852	n/a
			IMPACT without CT + Volumes		0,700	0,872	0,853	n/a
			IMPACT without CT + Shapes		0,682	0,868	0,841	n/a
			IMPACT without CT + Shapes + Volumes		0,734	0,852	0,85	n/a
			IMPACT lab model feature list (baseline)		0,677	0,829	0,819	n/a
			IMPACT without CT (baseline)		0,639	0,834	0,776	n/a
	AlexNet-CNN	Pease et al.	Internal (UPMC)		n/a	Fixed	0.86 (0.79–0.94)	n/a
			External (TRACK-TBI)		0,1	Fixed	0.83 (0.76–0.89)	n/a
6 to 12 months Based on GOSE class	AlexNet-CNN	Pease et al.	Internal (UPMC)	GOS 1–3	n/a	n/a	0,83 (0.75–0.92)	n/a
			External (TRACK-TBI)	GOS 1–3	0,08 (Sensitivity @ Spec 100%)	Fixed	0.73 (0.66–0.81)	n/a
				GOS 1–4	0,16 (Sensitivity @ Spec 95%)	Fixed		
				GOS 1–5	0,3 (Sensitivity @ Spec 90%)	Fixed		
	3D-ViT	Hibi et al.	Internal (CENTER-TBI)		0,774	0,777	0,846	n/a
			External (CINTER-TBI)		0,708	0,874	0,859	n/a
	MSF	Zhang et al.	Top1 prediction of GOSE class out of 8 classes		n/a	n/a	n/a	0,8406
			Top2 prediction of GOSE class out of 8 classes		n/a	n/a	n/a	0,9478
			Top3 prediction of GOSE class out of 8 classes		n/a	n/a	n/a	0,9813
Need for therapy: TILsum_High (≥ 11) vs. TILsum_Low (< 11)	CNN1: BLAST-CT	Brossard et al.	Internal		n/a	n/a	0,600	0,5
			External					0,67
	CNN2: 4-class segmentation	Brossard et al.	Internal		n/a	n/a	n/a	0,67
			External					0,67
	CNN3: 7-class segmentation	Brossard et al.	Internal		n/a	n/a	n/a	0,83
			External					0,75
	CNN4: 7-class segmentation	Brossard et al.	Internal		n/a	n/a	n/a	0,83
			External					0,83
14-day Death vs. Survival	LR	Xu et al.	CRASH-BASIC		0,858	n/a	n/a	0,7815
			CRASH-CT					0,7682
			CRASH-CT + Volumes					0,8011
			CRASH-CT + Volumes + Subtypes					0,8075
	RF	Xu et al.	CRASH-BASIC		0,769	n/a	n/a	0,7524
			CRASH-CT					0,8095
			CRASH-CT + Volumes					0,8
			CRASH-CT + Volumes + Subtypes					0,819
14-day Death and Coma vs. others	LR	Xu et al.	CRASH-BASIC		0,858	n/a	n/a	0,7742
			CRASH-CT					0,7746
			CRASH-CT + Volumes					0,8206
			CRASH-CT + Volumes + Subtypes					0,834
	RF	Xu et al.	CRASH-BASIC		0,769	n/a	n/a	0,7905
			CRASH-CT					0,819
			CRASH-CT + Volumes					0,8762
			CRASH-CT + Volumes + Subtypes					0,9048
Mass effect: Surgery required | not required	3D UNet DNN	Puzio et al.			0,745	0,877	0,88	n/a
High ICP (≥ 20 mmHg) vs. Low ICP (< 20 mmHg)	PY	Sato et al.			n/a	n/a	n/a	0,7727
	PO	Sato et al.			n/a	n/a	n/a	0,8409
	SR	Sato et al.			n/a	n/a	n/a	0,6136
3 months GOSE class	MSF	Zhang et al.	Top1 prediction of GOSE class out of 8 classes		n/a	n/a	n/a	0,8371
			Top2 prediction of GOSE class out of 8 classes		n/a	n/a	n/a	0,9464
			Top3 prediction of GOSE class out of 8 classes		n/a	n/a	n/a	0,9607
72 h surgical vs. no-surgical need	ViT	Smith et al.	Dataset 1 (*n* = 2806)		0,87	0,88	0,92	0,87
			Dataset 2 (*n* = 612)		0,85	0,84	0,89	0,84
Mortality vs. Survival	MLP	Wu et al.	0,4 Threshold		0,713	0,801	0,819	0,788
			0,5 Threshold		0,683	0,827		0,805
			0,6 Threshold		0,536	0,923		0,865
			0,7 Threshold		0,443	0,959		0,882

### Performance metrics of models utilized for prognosis outcome

Across studies that evaluated the prognosis outcome, prognostic performance demonstrated moderate to high discrimination, with substantial variability depending on outcome type. The results are summarized in Table 4.

#### Mortality prediction

For mortality-related outcomes, reported performance metrics varied across time horizons and modeling approaches. For 6-month mortality, mean AUC values ranged from approximately 0.778 to 0.86 [17, 31]. For short-term outcomes, including 14-day mortality [24] and combined death and coma outcomes [24], reported accuracy values ranged from approximately 0.75 to 0.90, with some models achieving accuracies above 0.80 when CT volumes and hemorrhage subtypes were included [17, 24]. Overall mortality prediction models also demonstrate moderate discrimination, with reported AUC values around 0.82 and accuracy values ranging from approximately 0.79 to 0.88, depending on decision thresholds [28].

#### Functional outcome

For functional outcome prediction, including GOSE-based outcomes, reported performance varied by outcome definition and time point. For multiclass GOSE prediction, a top-1 accuracy of approximately 0.84 was reported [35]. When evaluated using top-k accuracy, performance increased substantially, with top-2 and top-3 accuracy reaching approximately 0.95 and 0.98 [35]. This reflects the proportion of cases in which the true GOSE class was contained within the top one, two, or three most probable predictions ranked by the model for each input examination [35].

#### Surgical intervention, therapy intensity, and secondary injury markers

For prediction of neurosurgical intervention, reported AUC values were from 0.88 to 0.92, with sensitivity and specificity values from 0.85 to 0.88 [25, 36]. Prediction of therapy intensity level demonstrated more variable performance. Internal validation accuracy values ranged from approximately 0.50 to 0.83, with external validation accuracy values ranging from approximately 0.67 to 0.83 [32]. For prediction of high versus low intracranial pressure, reported accuracy values ranged from approximately 0.61 to 0.84, depending on model type and feature sets [33].

#### Models training, validation, and external testing for prognosis outcome tasks

Training and validation strategies for prognostic models varied across studies, with most relying primarily on internal validation employing fixed train–test splits or k-fold cross-validation (Supplementary Table S2). These approaches were used in studies predicting functional outcome, as well as in some mortality and secondary injury prediction models [17, 24, 25, 28, 31, 33].

Only four (4) studies incorporated independent external validation [31, 32, 34, 36]. External validation for mortality and functional outcome prediction was reported by Pease et al. [31], using the TRACK-TBI external cohort, and by Hibi et al. [34], using external CENTER-TBI/CINTER-TBI cohorts.

For therapy intensity prediction, Brossard et al. evaluated model performance using both internal and external datasets [32]. For prediction of neurosurgical intervention, Smith et al. evaluated model performance across two independent datasets, providing an additional form of external assessment [36].

Across studies that included external validation, prognostic performance was variable and, in several cases, lower than that reported in internal cohorts, indicating reduced generalizability and potential overfitting [31, 32]. This pattern was particularly evident for functional outcome and mortality prediction.

Reporting of training, validation, and testing procedures was inconsistent across prognostic studies. In addition, sample sizes for prognostic model development and testing varied widely, with some studies relying on relatively small validation or test cohorts [28].

## Discussion

In this systematic review, we identified a wide range of AI-based models developed for both diagnostic and prognostic tasks in patients with TBI using CT imaging. Overall, diagnostic models demonstrated generally high performance, particularly for intracranial hemorrhage detection, lesion segmentation, and midline shift assessment, whereas prognostic models showed more heterogeneous performance and were frequently limited by insufficient external validation and high risk of bias. These findings suggest substantial potential for AI-assisted decision support in acute neuroimaging, while also indicating that current evidence remains insufficient for widespread clinical implementation.

Our findings are in accordance with the systematic review by Hibi et al., which demonstrated that several clinically relevant CT features of TBI can be automatically identified and quantified using AI-based approaches [6]. These findings are clinically relevant because early therapeutic intervention and prompt transfer to a facility with neurosurgical capabilities are critical for optimizing outcomes in patients with TBI [37, 38]. Recent advances in AI-based image analysis may support this process by enabling automated detection of pathological CT findings [18–20, 23] and estimation of prognosis [17, 31, 33, 34]. In addition, large-scale datasets and multicenter databases have facilitated the development of algorithms for detecting and classifying intracranial hematoma and midline shift, an important radiological marker of elevated intracranial pressure [39].

These conclusions are further supported by a recent systematic review encompassing diagnostic, prognostic, and monitoring applications of AI in TBI, which reported promising discrimination across tasks but emphasized that clinical translation remains limited by retrospective designs, small cohorts, and inadequate external validation [40]. Similar methodological limitations have also been reported in pediatric TBI, where a recent systematic review of machine learning–based models demonstrated high diagnostic and prognostic performance for CT utilization and outcome prediction, but with minimal external validation and predominantly retrospective study designs [41].

However, despite these encouraging results, translation of these findings into routine clinical practice remains limited. The observed variability in model architectures, target tasks, and evaluation strategies reflects a rapidly evolving field but also underscores the current lack of standardization. Differences in reported performance between internal and external validation cohorts further suggest that real-world performance may be lower than that reported in controlled research settings.

Several studies demonstrated volume-dependent diagnostic performance, with substantially improved accuracy for larger hemorrhage volumes and lower performance for small-volume lesions. This suggests that current AI systems may be particularly effective for identifying clinically overt pathology, while further refinement is needed for reliable detection of subtle findings.

A key finding of this review is the limited use of independent external validation across both diagnostic and prognostic studies. Less than half of studies evaluated model performance on truly independent external datasets [18–20, 27, 29, 31, 32, 34, 36]. This finding underscores the importance of external validation as a critical requirement for assessing model transportability and real-world clinical reliability. Without adequate external validation, reported performance metrics may overestimate clinical effectiveness.

Furthermore, while several studies reported near-perfect diagnostic and prognostic performance, these results should be interpreted as indicators of technical feasibility rather than definitive evidence of clinical effectiveness. Future research should prioritize prospective, multicenter validation, standardized reporting, and evaluation within real-world clinical workflows to establish the true clinical impact of AI-based tools for automated TBI diagnosis based on CT imaging.

### Limitations

This study has several important limitations. A major limitation arises from the overall methodological quality of the included studies. As demonstrated by the QUADAS-2 and PROBAST + AI assessments, a substantial proportion of studies were at high risk of bias, particularly in the patient selection domain. Although applicability concerns were generally low for index tests, predictors, and outcomes, several studies demonstrated limited applicability due to population-related issues. This may increase the likelihood of spectrum bias and limit the representativeness of the studied populations.

In addition, all prediction model studies evaluated with PROBAST + AI were judged to be at high overall risk of bias, primarily due to methodological shortcomings in the analysis domain. Common issues included inadequate handling of missing data, insufficient reporting of model development procedures, limited or absent external validation, and potential overfitting. These deficiencies may lead to overly optimistic performance estimates and reduce confidence in the reproducibility and generalizability of reported results.

One other limitation is that all included studies were retrospective in design and employed a wide range of AI model architectures, input features, target tasks, and outcome definitions. There was marked heterogeneity in training, validation, and testing strategies, as well as in the datasets used for model development. This methodological diversity limits direct comparison across studies and reduces confidence in the generalizability of reported performance.

Another limitation relates to the datasets used for model development and training. Some studies relied on publicly available hemorrhage datasets that were not exclusively restricted to TBI populations. Consequently, some lesions used for model training and validation may have originated from non-traumatic etiologies, which may limit the generalizability of the reported performance to dedicated acute TBI populations.

Moreover, the high heterogeneity across studies precluded quantitative synthesis. Differences in patient populations, injury severity, CT acquisition and reconstruction protocols, reference standards, target definitions, prognostic and outcome measures prevented meaningful meta-analysis. As a result, conclusions are based on narrative synthesis rather than pooled effect estimates, which may reduce the precision of overall inferences.

Furthermore, publication and reporting biases cannot be excluded. Grey literature was excluded, and studies with negative or null findings may be underrepresented in the published literature. Finally, this review focused exclusively on studies using CT as the sole imaging modality. While this was intentional to maintain clinical relevance and methodological consistency, it excludes potentially informative multimodal approaches that integrate CT imaging with clinical data, laboratory parameters, or other imaging modalities. Therefore, the findings may not fully reflect the broader landscape of AI-based TBI assessment.

### Future directions

Although many studies report encouraging diagnostic and prognostic performance, the current evidence base is constrained by methodological weaknesses, substantial heterogeneity, and limited external validation. These factors should be considered before widespread clinical implementation of AI-based tools for TBI diagnosis and prognosis.

Future research should focus on prospective, multicenter studies using standardized imaging protocols, uniform outcome definitions, and consistent modeling strategies, with particular emphasis on rigorous external validation using independent datasets to better assess generalizability and real-world performance. In addition, the development of large multicenter TBI-specific CT databases would facilitate the training and validation of more robust AI models and improve their generalizability across different patient populations and healthcare settings. Notably, ongoing prospective studies (NCT06027411, AI-PROBE) [42], evaluating AI-based CT head prioritization in emergency workflows, and the RADIOMIC-TBI trial (NCT04058379) [43], assessing serial CT-based AI analysis for predicting lesion evolution and neurological outcomes, represent important steps toward translating AI tools from retrospective validation to routine clinical practice.

Further work should also evaluate the real-world clinical impact of AI tools, including their effects on workflow efficiency, time to diagnosis, interobserver variability, neurosurgical triage, and patient-centered outcomes. Ultimately, well-designed prospective implementation studies will be essential to determine whether AI-based systems can safely and effectively improve clinical decision-making and outcomes in patients with traumatic brain injury.

## Conclusion

This systematic review demonstrates that AI–based models applied to head/brain CT have achieved high technical performance for a wide range of diagnostic and prognostic tasks in TBI patients. Diagnostic applications, particularly for intracranial hemorrhage detection, lesion segmentation, and midline shift, appear to be more mature and consistently validated than prognostic applications.

In contrast, prognostic models for mortality, functional outcome, and secondary injury markers show greater heterogeneity in performance and remain limited by methodological weaknesses and insufficient external validation. Although many studies report promising results, the current evidence base is constrained by retrospective designs, limited transportability, and substantial variability in modeling and evaluation strategies.

Overall, while AI-based tools show considerable potential to support clinical decision-making, further prospective multicenter with extrenal validation are essential to establish their clinical utility, safety, and generalizability before widespread clinical adoption.

## Supplementary Information

Below is the link to the electronic supplementary material.

Search algorithm

Prisma check list

List of tables
Table S1. Models training, validation, and external testing for diagnostic tasks

Table S2. Models training, validation, and external testing for prediction outcome tasks

## Data Availability

No datasets were generated or analysed during the current study.

## Abbreviations

AI: Artificial Intelligence
ANN: Artificial Neural Network
AUC: Area Under the Curve
CNN: Convolutional Neural Network
CT: Computed Tomography
ΕΑΗ: Extra Axial Hemorrhage
EDH: Epidural Hematoma
GNN: Graph Neural Network
GOS: Glasgow Outcome Scale
GOSE: Glasgow Outcome Scale–Extended
ICP: Intracranial Pressure
ICH: Intracranial Hemorrhage
IMPACT: International Mission for Prognosis and Analysis of Clinical Trials in TBI
IPH: Intraparenchymal Hemorrhage
IVH: Intraventricular Hemorrhage
MLS: Midline Shift
N/A: Not Applicable
PROBAST: Prediction Model Risk of Bias Assessment Tool
PRISMA: Preferred Reporting Items for Systematic Reviews and Meta-Analyses
QUADAS-2: Quality Assessment of Diagnostic Accuracy Studies:2
SAH: Subarachnoid Hemorrhage
SDH: Subdural Hematoma
TBI: Traumatic Brain Injury
WHO: World Health Organization
