# Supplementary material for: The role of artificial intelligence in the diagnosis and prognosis of traumatic brain injury based on brain CT scans: a systematic review
Source: Neurosurg Rev. 2026 Sep 12;49(1):561. doi: 10.1007/s10143-026-04476-7 (PMC13569537; doi:10.1007/s10143-026-04476-7)
Supplement: Supplementary file 1 [file 10143_2026_4476_MOESM1_ESM.docx]

**Search Algorithm for each database**

1. **Pubmed**

("deep learning" OR "deep neural network" OR "DNN" OR "neural network" OR "artificial neural network" OR "ANN" OR "convolutional neural network" OR "CNN" OR "recurrent neural network" OR "RNN" OR "long short-term memory" OR "LSTM" OR "transformer" OR "transformer model")

AND

("traumatic brain injury" OR TBI OR "brain trauma" OR "head injury")

AND

(MRI OR "magnetic resonance imaging" OR CT OR "computed tomography" OR "head CT" OR "brain scan" OR neuroimaging OR radiomics OR "functional MRI" OR "fMRI" OR "diffusion tensor imaging" OR "DTI" OR "PET" OR "PET imaging")

AND

(prognosis OR prediction OR predict OR classifier OR classify OR classification OR categorization OR "risk stratification" OR "severity grading" OR triage OR "surgical decision" OR "intervention decision" OR "clinical outcome" OR "outcome prediction")

NOT

(review[Publication Type])

2. **Scopus**

(TITLE-ABS-KEY("deep learning" OR "neural network" OR "convolutional neural network" OR "CNN" OR "recurrent neural network" OR "RNN" OR "LSTM" OR "transformer" OR "deep neural network" OR "DNN"))

AND

(TITLE-ABS-KEY("traumatic brain injury" OR TBI OR "brain trauma" OR "head injury"))

AND

(TITLE-ABS-KEY(MRI OR "magnetic resonance imaging" OR CT OR "computed tomography" OR neuroimaging OR radiomics OR "functional MRI" OR "fMRI" OR "diffusion tensor imaging" OR "DTI" OR "PET imaging"))

AND

(TITLE-ABS-KEY(prognosis OR prediction OR predict OR classification OR classify OR "risk stratification" OR triage OR "surgical decision" OR "clinical outcome"))

3. **IEEE Xplore**

("deep learning" OR "neural network" OR "CNN" OR "convolutional neural network" OR "RNN" OR "LSTM" OR "transformer" OR "deep neural network" OR "DNN")

AND

("traumatic brain injury" OR TBI OR "brain trauma" OR "head injury")

AND

(MRI OR "magnetic resonance imaging" OR CT OR "computed tomography" OR "brain imaging" OR neuroimaging OR radiomics OR "functional MRI" OR "diffusion tensor imaging" OR "DTI" OR PET)

AND

(prognosis OR prediction OR predict OR classification OR triage OR "clinical outcome")

4. **ACM Digital Library**

("deep learning" OR "neural network" OR "CNN" OR "convolutional neural network" OR "RNN" OR "LSTM" OR "transformer" OR "deep neural network" OR "DNN")

AND

("traumatic brain injury" OR TBI OR "brain trauma" OR "head injury")

AND

(MRI OR "magnetic resonance imaging" OR CT OR "computed tomography" OR "brain imaging" OR neuroimaging OR radiomics OR "functional MRI" OR "diffusion tensor imaging" OR "DTI" OR PET)

AND

(prognosis OR prediction OR predict OR classification OR triage OR "clinical outcome")

5. **Cochrane**

("traumatic brain injury" OR TBI) AND

("deep learning" OR "neural network" OR "convolutional neural network" OR "CNN" OR "machine learning") AND

(MRI OR "CT scan" OR neuroimaging OR radiomics) AND

(predict OR prognosis OR classification OR "treatment decision")
