# Supplementary material for: The role of artificial intelligence in the diagnosis and prognosis of traumatic brain injury based on brain CT scans: a systematic review
Source: Neurosurg Rev. 2026 Sep 12;49(1):561. doi: 10.1007/s10143-026-04476-7 (PMC13569537; doi:10.1007/s10143-026-04476-7)
Supplement: Supplementary file 3 [file 10143_2026_4476_MOESM3_ESM.docx]

**Table S1: Models training, validation, and external testing for diagnostic tasks**

| **Author** | **Training** | **Validation** | **Testing** | **External Validation** |
| --- | --- | --- | --- | --- |
| Keshavamurthy et. al. | 327 CT scans (80%) | | 82 CT scans (20%) | No |
| Li et al. | 835 CT scans | 10-fold CV 180 CT scans | 179 CT scans | No |
| Yao et al. |  | 5-fold CV |  | No |
|  | 100 CT scans | | 20 CT scans |  |
| Monteiro et al. | 282 CT scans (Dataset 1-98 \| Dataset 2-184) | | 655 CT scans | Yes |
| Phaphuangwittayakul et al. | (EfficientNet-B2-15956 + DeepMedic, PhysioNET-2233) 18189 CT slices | | 4571 CT slices | Yes |
| Dyer et al. | 2059 CT scans | 390 CT scans | | Yes |
| Angkurawaranon et al. | Previous Study | | 301 CT scans | No |
| Vidhya et al. | 9552 CT scans (60%) | 3184 CT scans (20%) | 3185 CT scans (20%) | No |
| D'Angelo et al. | Pretrained | | 503 CT scans | No |
| Xu et al. | n/a | 5-fold CV | n/a | No |
|  | 151 CT scans | | |  |
| Puzio et al. | 189 CT scans | 85 CT scans | n/a | No |
| Sharma et al. | n/a | n/a | n/a | No |
| Lin et al. | 457 CT scans (Labeled) >25,000 CT scans (Unlabeled) | 93 CT scans | 1010 CT scans (481 + 529) | Yes |
| Wu et al. | 45 CT scans | 5 CT scans | 19 CT scans | No |
| Nagaraju et al. | 80% 20% Dataset 1: 82 CT scans | | n/a | Yes |
|  | 80% Dataset 2: 936 CT + MRI scans | | 20% Dataset 2: 936 CT + MRI scans |  |
|  | 80% Dataset 3: 7032 CT scans | | 20% Dataset 3: 7032 CT scans |  |
| Emon et al. | 10% Randomly selected 82 CT scans | | | No |
