# Supplementary material for: The role of artificial intelligence in the diagnosis and prognosis of traumatic brain injury based on brain CT scans: a systematic review
Source: Neurosurg Rev. 2026 Sep 12;49(1):561. doi: 10.1007/s10143-026-04476-7 (PMC13569537; doi:10.1007/s10143-026-04476-7)
Supplement: Supplementary file 4 [file 10143_2026_4476_MOESM4_ESM.docx]

**Table S2: Models training, validation, and external testing for prognosis outcome tasks**

| **Author** | **Training** | **Validation** | **Testing** | **External Validation** |
| --- | --- | --- | --- | --- |
| Yao et al. | 10-fold CV 828 CT scans | | | No |
| Pease et al. | 70% | 10% | 108 CT scans (20%) | Yes |
|  | 537 CT scans | | |  |
| Brossard et al. | 60% | 20% | 20% | Yes |
|  | 84 CT scans | | |  |
| Xu et al. | n/a | 5-fold CV | n/a | No |
|  | 151 CT scans | | |  |
| Puzio et al. | 189 CT scans | 85 CT scans | n/a | No |
| Sato et al. | 168 CT slices | 42 CT slices | 44 CT slices | No |
|  | 77 CT scans | | |  |
| Hibi et al. | 80% | 5-fold cross CV  20% | | Yes |
|  | 1016 CT scans | | |  |
| Zhang et al. | appr. 75% | appr. 25% | | No |
|  | 650 CT scans | | |  |
| Smith et al. | 2244 CT scans | | 562 CT scans | Yes |
| Wu et al. | n/a | n/a | n/a | No |
